# Rhythms and synchronization patterns in gene expression in the *Aedes aegypti *mosquito

**DOI:** 10.1186/1471-2164-12-153

**Published:** 2011-03-17

**Authors:** Andrey A Ptitsyn, Guadalupe Reyes-Solis, Karla Saavedra-Rodriguez, Jonathan Betz, Erica L Suchman, Jonathan O Carlson, William C Black

**Affiliations:** 1 Center for Bioinformatics, Colorado State University, Fort Collins, CO 80525, USA; 2 Department of Microbiology, Immunology and Pathology, College of Veterinary and Biomedical Sciences, Colorado State University, Fort Collins, CO 80525, USA

## Abstract

**Background:**

*Aedes aegypti *is arguably the most studied of all mosquito species in the laboratory and is the primary vector of both Dengue and Yellow Fever flaviviruses in the field. A large number of transcriptional studies have been made in the species and these usually report transcript quantities observed at a certain age or stage of development. However, circadian oscillation is an important characteristic of gene expression in many animals and plants, modulating both their physiology and behavior. Circadian gene expression in mosquito species has been previously reported but for only a few genes directly involved in the function of the molecular clock.

**Results:**

Herein we analyze the transcription profiles of 21,494 messenger RNAs using an *Ae. aegypti *Agilent^® ^microarray. Transcripts were quantified in adult female heads at 24 hours and then again at 72 hours and eight subsequent time points spaced four hours apart. We document circadian rhythms in multiple molecular pathways essential for growth, development, immune response, detoxification/pesticide resistance. Circadian rhythms were also noted in ribosomal protein genes used for normalization in reverse transcribed PCR (RT-PCR) to determine transcript abundance. We report pervasive oscillations and intricate synchronization patterns relevant to all known biological pathways.

**Conclusion:**

These results argue strongly that transcriptional analyses either need to be made over time periods rather than confining analyses to a single time point or development stage or exceptional care needs to be made to synchronize all mosquitoes to be analyzed and compared among treatment groups.

## Background

Mosquito species exhibit a wide range of distinct daily activity patterns and are, in fact, frequently characterized as to whether their feeding and mating behaviors are diurnal, crepuscular, or nocturnal (reviewed in [1] chapter 33). Activity and blood-feeding rhythms appear to be controlled by a circadian (i.e. approximately daily) clock [1]. Observations of circadian rhythms in the physiology and behavior of the culicine mosquito species *Aedes aegypti *(L) have been previously reported in the field (biting patterns [2], biting and flight patterns [3]) and in laboratory studies (oviposition [4]; sugar-feeding [5]). Both oviposition and sugar-feeding patterns have been explained by an inherited endogenous circadian rhythm [1]. Entraining of flight pattern caused by the change from light to dark as a time-cue was experimentally studied by Taylor and Jones[6]. Subsequent publications in the culicine mosquito *Culiseta incidens *reported differences in behavior in constant darkness and constant light after light/dark 12 h:12 h entraining. The authors proposed the existence of two dependent oscillators which could be temporarily or permanently uncoupled to explain lengthening and shortening of activity periods [7,8]. Connection between metabolism, feeding pattern and circadian regulation of gene expression has been explored by Das and Dimopoulos [9]. This study taking advantage of custom designed microarrays has reported that genes controlling feeding behavior are under circadian control. Long and short light pulses can alter circadian feeding behavior through unknown molecular mechanisms, possibly involving chemosensory system [9].

In no other insect species have the genes that control circadian rhythms been as well characterized as in *Drosophila melanogaster*. A number of genes have been implicated in the control of circadian rhythms in this species. Transcriptional analyses of these suggest a mechanism whereby transcriptional negative feedback loops control cyclic expression [10]. The two main regulatory loops of the circadian clock involve the genes *period *(*per*), *timeless *(*tim*), *Clock *(*Clk*), *cycle *(*cyc*), *vrille *(*vri*), and *Par-domain-protein-1 *(*Pdp1*) [11-13]. Regulatory loops control the circadian expression of genes in the clock and determine the abundance of a large number of fluctuating transcripts. These transcripts in turn are thought to be indicators of a pacemaker that controls various aspects of physiology and behavior [14].

Orthologues of *Drosophila *circadian genes have been cloned or identified in the mosquitoes *Anopheles gambiae *[15] and *Ae. aegypti *[16]. Gentile et al [17] cloned and analyzed expression of *tim *in *Ae. aegypti*. In *Drosophila, tim *controls a central pacemaker and a resetting mechanism that allows the clock to synchronize with environmental light-dark cycles. Predicted protein sequence encoded by *timeless *in *Ae. aegypti *and *D. melanogaster *were highly similar in domains of known function, suggesting functional conservation. Analysis of the daily expression of timeless indicated a peak in mRNA abundance around the daily light-dark transition. Gentile et al [18] compared the circadian expression of clock genes in *Ae. aegypti *and *Cx. quinquefasciatus*. Both species diverged > 22 MYA but exhibit conserved circadian expression patterns for all major cycling clock genes (with the exception of cryptochrome 2). However, beyond these few core circadian genes, little is known about circadian oscillations in gene expression in mosquitoes. Understanding these oscillations is essential for learning the relationship between the circadian clock and the observed periodicity in mosquito physiology and behavior.

Due to the ease with which it can be collected in the field and maintained in the laboratory, *Ae. aegypti *is the most studied of all mosquito species. Much of what we know about the genetic, biochemical, physiological, and behavior of mosquitoes has come from this species. For example, A.N. Clements' (1992) encyclopedia on mosquito physiology contains 800 references and 400 of these involve *Ae. Aegypti *[1]. As importantly the species is the principal vector of the dengue and yellow fever flaviviruses and a host of other important arboviruses throughout all tropical and subtropical regions of the world. Many transcriptional studies of genes involved in development [19,20], competence for pathogen propagation and transmission [21,22], insecticide resistance [23,24], blood-feeding [25] and blood meal digestion [26] have been conducted in *Ae. aegypti*. More recently, the species has also been the subject of a number of large scale transcriptional analyses using microarrays to scan for genes involved in insecticide resistance [27,28], development [29], vector competence [30-34], and bloodfeeding [35]. In no case did these studies (including those in which the senior author was directly involved) consider the possibility of circadian oscillations in expression of genes under study.

Herein we present a study in which we tested for circadian oscillations in expression of genes in a large part of the transcriptome using the Agilent^® ^*Aedes aegypti *microarray [15,33] and an experimental design that has been successfully used to demonstrate circadian cycles of gene transcription in model eukaryotic organisms (mouse, yeast) and humans. Transcripts were collected from the heads of females that were 72, 76, 80, 84, 88, 92, 96, 100 and 104 hours old. Transcripts were only collected from the heads of females for three reasons. First, whole bodies were not used because various studies have demonstrated asynchronous oscillations in transcript abundance among different organs and tissues. Analysis of whole carcasses could therefore mask tissue- or organ-specific oscillations in transcript abundance. Second, the head is a discrete structure that can be easily and rapidly dissected from the body without large amounts of contamination by transcripts from other tissues. We are of course assuming uniformity in transcript abundance among different organs and tissues in the head; an assumption that is almost certainly false. Third, we wished to avoid gender differences in transcript abundance. We demonstrate that a large number of transcripts in the female head exhibit circadian fluctuations.

## Results and discussion

**Figure 1:**
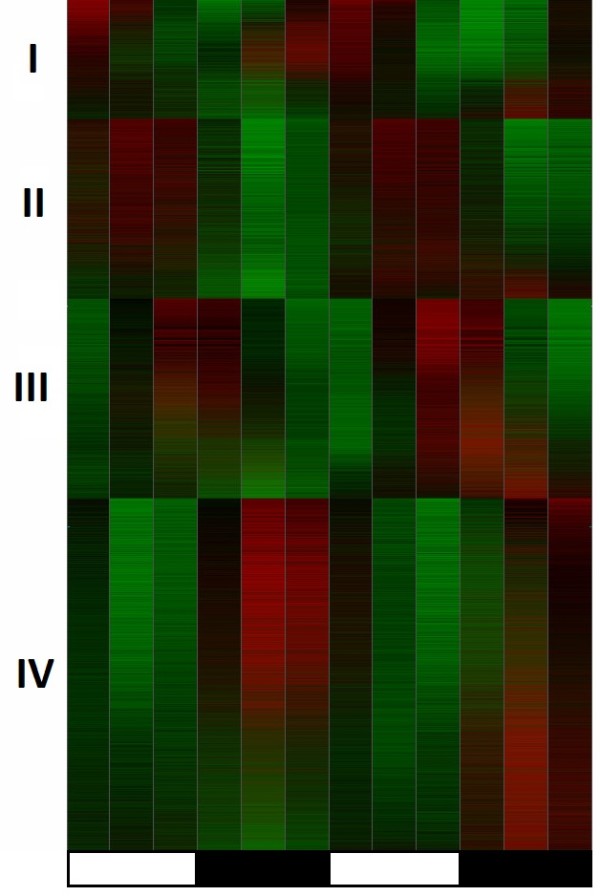
**Heatmap overview of the circadian pattern in *A. aegypti *transcriptome**. Red areas correspond to higher levels; green areas correspond to lower levels of expression over 2 days of observation. The succession of light and dark periods is marked along the bottom. Each expression profile is assigned one of four phases (marked on the left margin). The algorithm for generation of heatmap of gene expression is explained in Supplemental Figure 1 (see Additional File 1).

The amounts of the 21,494 unique transcripts on the Agilent^® ^*Aedes aegypti *microarray were quantified in adult female heads. The first time point was at 72 hours post-eclosion and each subsequent time point was collected every four hours for the next 32 hours. A heatmap of the female head transcriptome over 24 hours is presented in Figure 1. The heatmap shows a clear pattern of two peaks (red) and two troughs (green) in levels of gene expression over two days of observation, which corresponds to two 24 hour circadian cycles. This pattern is very similar to a previously published circadian heatmap of the murine transcriptome [36].

Figure 1 is also reminiscent of the heatmap diagram of periodic genes in *D. melanogaster *[37-39] with a few important differences. First, Figure 1 presents the entire female head transcriptome rather than pre-selected fraction of periodic transcripts. Second, we detected four groups of same-phase expression profiles rather than a rolling wave of profiles sorted by acrophase (the time of highest expression level). We assigned expression profiles to a phase group by fitting discrete expression profiles with low sampling rate (12 time points per series) to a discrete cosine curve generated with the same sampling rate. Hence, discrete presentation of four possible phases is the correct way to present the results.

The majority of transcripts in Figure 1 exhibit two cycles of oscillations over two days. This observation is consistent with the hypothesis that there is a gradual reduction of signal to noise ratio without an actual loss of periodicity [36]. Tests for periodicity applied in a straightforward way and testing each expression profile independently report considerable numbers of periodic transcripts (Table 1). The difference in estimated numbers of transcripts passing the standard cutoff of *p *= 0.05 is caused by the different set of assumptions underlying each of the tests. Application of statistical tests in a phase continuum setting mitigates some of the critical limitations of periodicity testing and allows improved signal to noise ratio by digital signal processing [40]. The numbers of periodic transcripts reported by tests in phase continuum (Table 1) are much higher and seem to be in a better agreement with the visual pattern (Figure 1) than numbers of rhythmically expressed genes reported in single-gene testing. Even the most conservative estimates reveal more cycling genes than previously reported in circadian expression profiling of *D. melanogaster*. Our experiment does not focus specifically on the genes controlled by circadian molecular clock; we register all genes expressed in a rhythmic pattern in LD environment. Some of the oscillations we observe may be harder to detect in altered light conditions. Although there must be some inter-species differences, we believe that most difference arise from the analysis methodology. The panel of statistical tests used in these studies applied to publicly available circadian expression profiles from the Gene Expression Omnibus (GEO) database consistently reports more cycling transcripts than the original publications [41]. It is also important to note that results of different tests presented on Table 1 are different estimations rather than exact count. It is reasonable to assume that among genes tested positive for circadian periodicity there are some false-positive results. In one of the earlier publications Ptitsyn et al. proposed a computational experiment with random permutation of time points in microarray circadian expression profiles [36]. One of the outcomes of that experiment was estimation of false-positive rate in straight application of Pt-test to one expression profile at a time. With all periodicity scrambled by random permutations about 10% of expression profiles still tested positive for baseline circadian rhythm. In our *Aedes aegypti *microarray analysis some of the expression profiles estimated as rhythmic may turn out false-positive in separate validation experiments. However, this number is likely to be less than 10%. Highly expressed transcripts have more favorite signal to noise ratio and less likely to produce false-positive results in periodicity tests. On the other hand, it would be incorrect to assume that transcripts that didn't pass the arbitrary p = 0.05 cutoff in tests are non-periodic. There are also false-negative rhythmically expressed transcripts, particularly among low-expressed genes, for which test are not sufficiently powerful. Reproducing the experiment with more time points (and thus higher sampling rate) can improve both testing for periodicity and determining the phase of oscillation. However, there are ways to improve the validity of existing data. First, selected microarray expression profiles can be validated using alternative RT-PCR technique for estimation of transcript abundance. Second, validity of gene expression patterns in time can be considered in the context of their interaction, i.e. within corresponding biological pathways.

**Table 1:** Numbers of rhythmically expressed (circadian) genes reported by statistical tests for periodicity

	Direct test	Phase continuum test
Fisher's g-test	8445 (19%)	38%
Pt-test	19067 (42%)	62%
Autocorrelation	6058 (13%)	83%

Total number of probes 45220		

All tests used standard p = 0.05 significance cutoff

**Figure 2:**
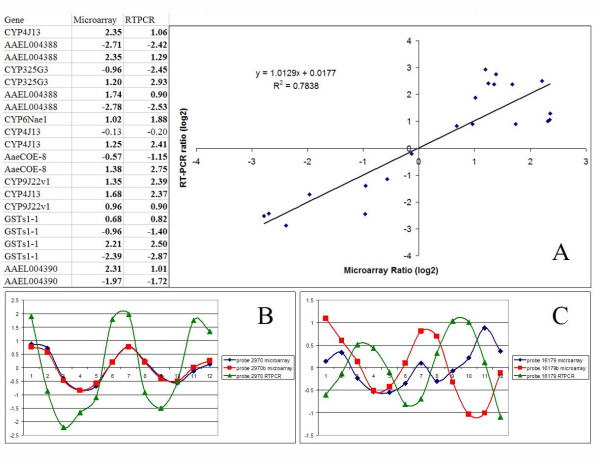
**Validation of microarray expression by RT-PCR**. A. Correlation between microarray significant ratios (log2) and average RTPCR ratio values. B. Timeline for microarray (two probes) and RTPCR expression values for AMP-dependent ligase. C. Timeline expression values for the hypothetic protein probe 16179 microarray (two probes) and RTPCR expression values.

Selected expression profiles were validated using RT-PCR (Figure 2). Overall the RT-PCR profiles are in a good agreement with the microarray profiles (panel A). Panel B shows agreement between intensity signal from microarray probes (two probes) and RT-PCR estimation of gene expression. For compatibility, raw RT-PCR values have been subtracted from the maximum. On the other hand, one of the microarray probes selected for RT-PCR validation demonstrated a clear circadian oscillating profile, but in a counter-phase to microarray expression profile (panel C). In turn, microarray probes are not in complete agreement with each other. The first probe (blue line) shows a mixed profile, which could be resulting from competing cross-hybridization between different transcripts. In previous publications it has been reported that alternatively polyadenylated transcripts of the same gene can oscillate in counter-phase to one another [42]. We hypothesize that RT-PCR expression values show behavior of only one of the alternative transcripts (green line), while microarray probes target the alternative transcript (probe b, red line) and both transcripts (blue line).

**Figure 3:**
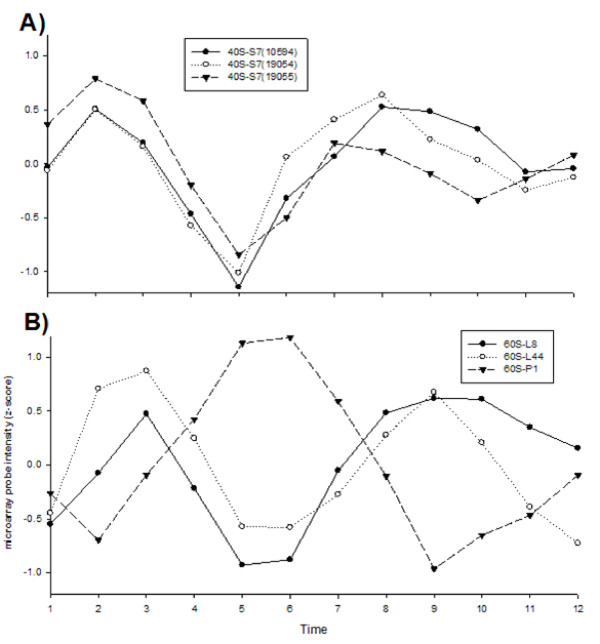
**Changes in the abundance of transcripts encoding the A) 40S ribosomal protein S7 and B) the 60S ribosomal proteins L8, L44, and P1**.

Genes encoding the 40S ribosomal protein S7 and the 60S ribosomal proteins L8, L44, and P1 are commonly used as "standards" for adjusting transcript abundance during RT-PCR. Figure 3a demonstrates clear and reproducible oscillations in 40S ribosomal S7 transcript abundance among the three probes for this gene in the Agilent^® ^Array. Figure 3b demonstrates clear oscillations in 60S ribosomal transcript abundance for L8 (UniGene:XM_001657661), L44 (UniGene: XM_001648014), and P1 (UniGene: XM_001656376). Note also that while 60S L8 and L44 transcripts are in the same phase that 60S-P1 is in opposite phase. Thus, conflicting signals would be obtained if one were to use 60S-P1 transcript abundance with abundance of any of the other three genes.

A general observation is that more highly transcribed genes tend to have less noisy expression profiles and instead exhibit a clear cycling profile. This is also consistent with the idea that the majority, if not all expressed genes experience diurnal variation in baseline expression. Thus we should account for such diurnal oscillation when considering gene function in the context of biological pathways. Coordinated non-random timing of peak and troughs of gene expression activity within a functionally related group can also serve as evidence of rhythmic expression.

**Figure 4:**
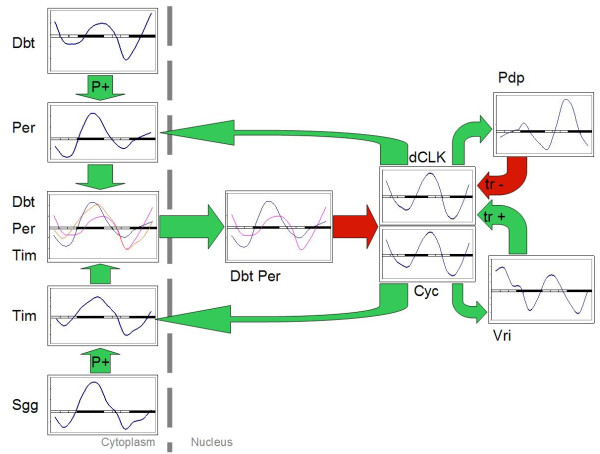
**Expression profiles of the components of circadian molecular clock of *A. aegypti***. The schema is adopted from KEGG database. Each pane shows expression profile of genes (marked by gene symbols) over two complete circadian periods. Light and dark periods are marked on the horizontal axis; vertical axis corresponds to relative abundance of transcripts (see Methods). Green arrows mark positive regulation, red arrows mark negative regulation of gene expression. P+ marks activation by phosphorylation, tr+ and tr- mark positive and negative regulation of transcription.

**Figure 5:**
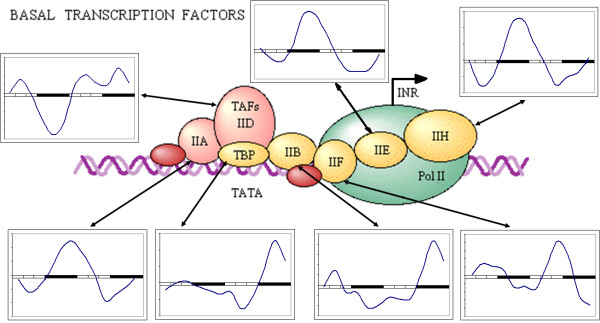
**Expression profiles of the basal transcription factors in *A. aegypti***. Elements of basal transcription complex show various degree of rhythmicity and different phase shifts modulating gene expression patterns. Each pane shows expression profile of genes (marked by gene symbols) over two complete circadian periods. Light and dark periods are marked on the horizontal axis; vertical axis corresponds to relative abundance of transcripts (see Methods).

The first example of coordinated rhythmic pattern in a biological pathway is presented in Figure 4. Here we present the components of the basic circadian clock identified in the mosquito genome form a circuit of the same design as the *D. melanogaster *molecular clock. The timeline expression profiles reported in Drosophila [43] are similar for the genes forming circadian molecular clock. In our experiment all components of the pathway are found to be oscillating and have expected differences in different phases of the oscillation. This observation confirms the previous report of circadian oscillation in A. aegypti mosquito [9]. In contrast to that study we did not attempt to alter the pattern of gene expression by alternation in light condition. Instead we used the same type of microarrays to probe longer time spans with regular sampling. This design allowed identification of oscillation and phase shift with more precision in much larger number of expressed genes. The fact that the majority of expressed genes experience circadian oscillation also means that oscillation affects the majority of biological pathways. Some of the pathways implicated in oscillatory expression are discussed in Das and Dimopoulos report [9]. We confirm the original report and extrapolate the oscillatory pattern to all other pathways. Evidence of coordinated timing of gene expression is seen in Figure 5, showing the expression profile of the mosquito basal promoter complex. All elements show some evidence of periodicity, but transcription factors TFIIA [UniGene: XM_001652453] and TFIIH [UniGene:XM_001651873] are expressed clearly in counter-phase to transcription factors TFIIF [UniGene:XM_001648018] and TFIID [Unigene: XM_001657785]. Previously reported differences in oscillation patterns of basal transcription factors in murine bone [44] has been attributed to differential modulation of gene expression by different signaling systems, creating complex orchestration of transcription for different genes. In *A. aegypti *the pattern might be less complicated or the analysis might have omitted minor details due to technical noise. Overall, parts of the basal transcription complex and transcription factors in particular, follow the pattern set by the elements of the basic molecular clock, such as TFIID-F expressed in the same phase as Pdp and TFIIA in the same phase with dCLK [UniGene: XM_001654547] (opposite to Pdp, [UniGene: XM_001650542]). Timeless (Tim, [UniGene: XM_001657734]) and Period (Per, [Unigene: XM_001658926]) oscillate in the same phase with TFIIH slightly ahead of dCLK and Cycle (Cyc, [XM_001654547]). Among all pathways composed of oscillating genes basic energy metabolism is particularly significant.

**Figure 6:**
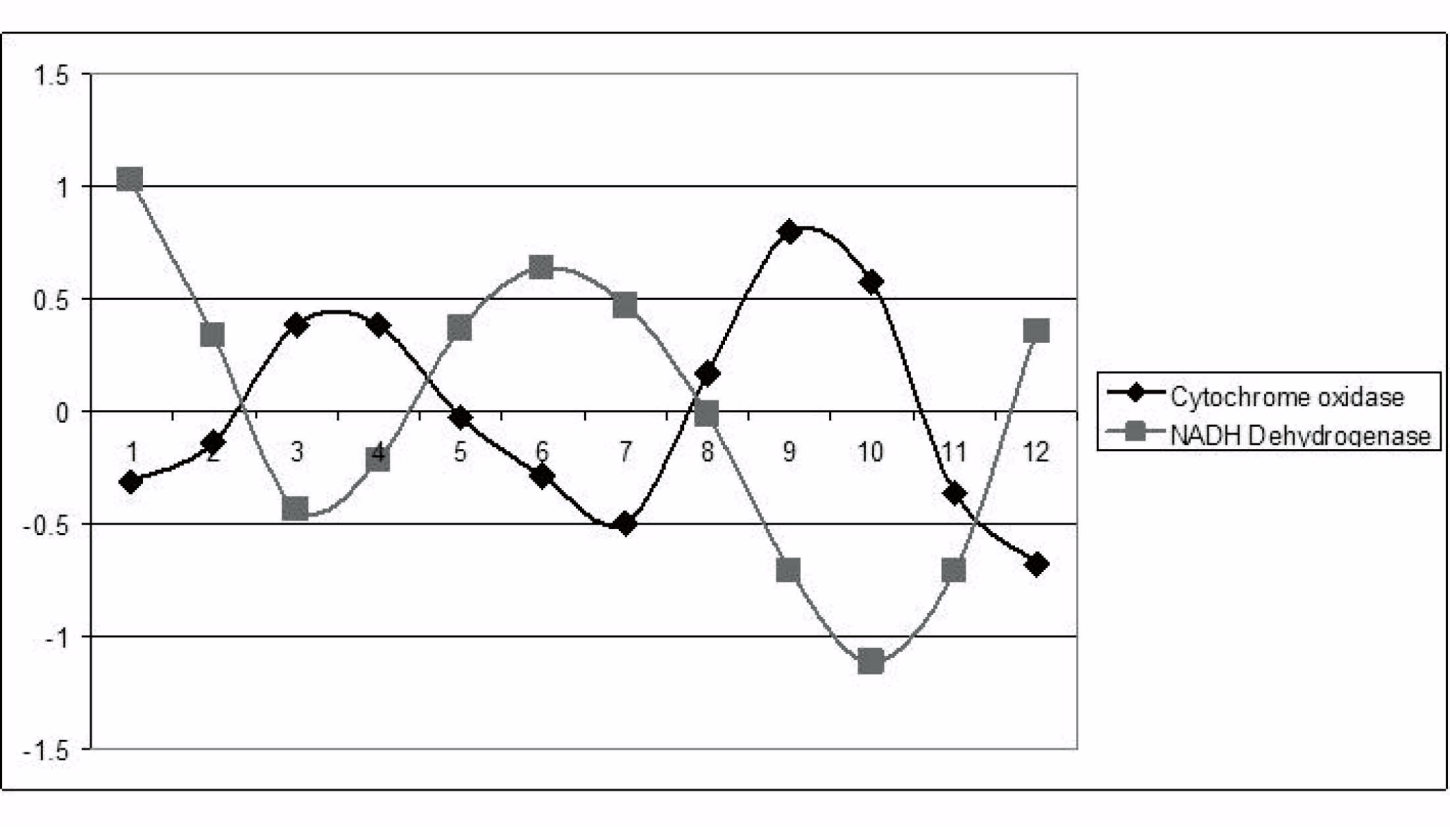
**Expression profiles of genes in oxidative phosphorylation pathway**. Ordinate shows relative abundance (z-score of microarray probe intensity) in 12 time points (abscissa) evenly spaced over 2 days of observation.

The expression profiles of the major components of oxidative phosphorylation are presented in Figure 6. Remarkably, all genes involved in oxidative phosphorylation are among the most prominently oscillating genes. Phase shift in production rates for the oxidative phosphorylation genes is in agreement with expectations from the general model of the process: components of the oxidative phase are expressed in counter-phase to the components of the reductive phase. Previous studies have pointed to the oxidative phosphorylation pathways as the intrinsic oscillator [45] modulating expression of many genes [46,47] and gating DNA replication [48]. Other studies outline the regulatory connection between basic metabolism and circadian clock [49]. Even though these studies used different model organisms it is reasonable to assume a connection between circadian clock and oxidative phosphorylation in mosquitoes as well. We hypothesize that circadian clock and oxidative phosphorylation are two main intrinsic oscillators modulating physiology and behavior of *A. aegypti *mosquitoes. These oscillators are linked and work in synchrony, but can be temporarily or permanently uncoupled by changing environmental conditions or due to mutations that lead to the creation of the behavioral patterns reported in previous publications [7].

Understanding circadian oscillation rhythms and gene synchronization patterns is essential to understanding mosquito molecular biology. Pesticide resistance is closely related to detoxification pathways which in turn are interlinked with oxidative phosphorylation. The oscillating pattern of oxidative phosphorylation gene expression may modulate the mosquito's ability to withstand insecticide exposure. On the other hand a mosquito's metabolism is also modified by a blood meal, which changes the energy balance from glycolytic to oxidative phosphorylation, changing the synchronous pattern for multiple genes. This alteration in expression timing can be potentially used to identify the age of larvae and adults, the availability of blood meal and other parameters important for disease control. There is a reason to believe that egg formation in mosquitoes is also gated by oxidative phosphorylation in a way similar to DNA replication in yeast [48] thus minimizing DNA damage from free radicals. Understanding the mechanisms of gene orchestration and correct timing of gene expression may help to identify the most vulnerable times and most promising targets for intervention in ever shifting patterns of interacting genes networks.

**Figure 7:**
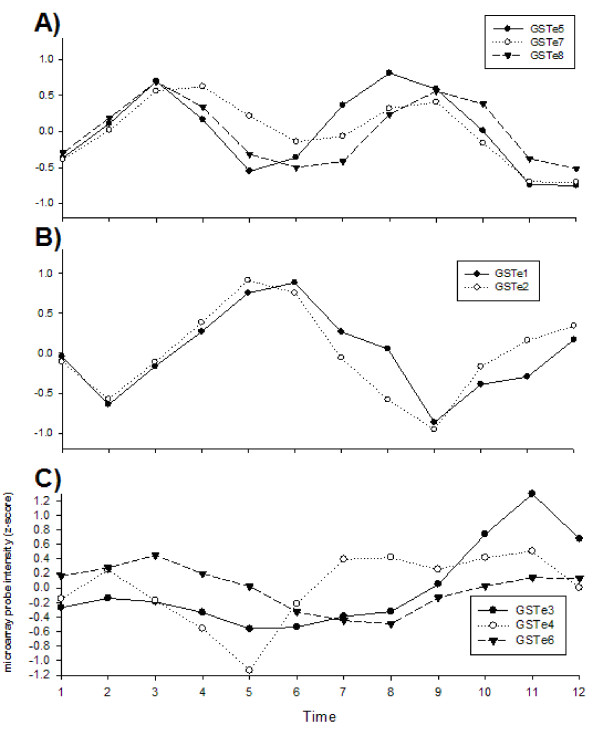
**Shifts in the abundance of transcripts for epsilon-class Glutathione-S-transferases (GST) genes 1-8**.

**Figure 8:**
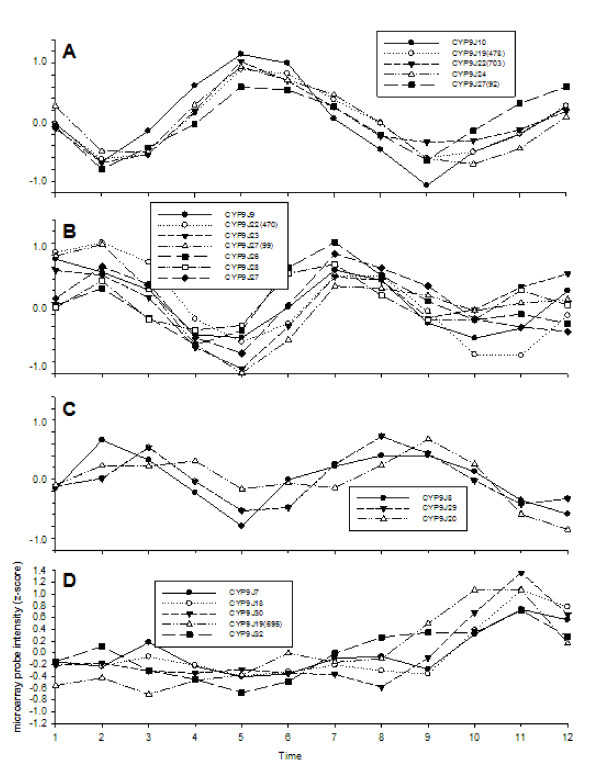
**Shifts in the abundance of transcripts 17 members of the CYP9J family of cytochrome P450 superfamily of genes CYP9J family**.

We also examined expression of genes involved in metabolism of xenobiotics in general and insecticides in particular (Figures 7 and 8). Glutathione-S-transferases (GST [Unigene: XM_001648685]) are a diverse family of enzymes involved in a wide range of biological processes, many of which involve the conjugation of the tripeptide glutathione to an electrophilic substrate. Elevated levels of GST activity have been associated with resistance to all the major classes of insecticides [50]. The epsilon-class GSTs in particular are frequently associated with resistance. Figure 7 examined the abundance of transcripts from the eight epsilon class GSTs. All of these are located in a single cluster on chromosome 2 in the *Ae. aegypti *genome[51,27]. Figure 7A demonstrates co-oscillation of GST -5 [VectorBase: AAEL007964], GST -7 [VectorBase: AAEL007948], and GST -8 [VectorBase: AAEL007955] transcript abundances while Figure 7B shows co-oscillation of GST -1 [VectorBase: AAEL00795] and GST -2 [VectorBase: AAEL007951] transcript abundances. Figure 7C demonstrates that GST -3 [VectorBase:AAEL007947], GST -4 [VectorBase:AAEL007962] and GST -6 [VectorBase:AAEL007946] transcripts do not co-oscillate with the other GST in Figures 7a and 7b nor with one another. GST -6 shows only shallow oscillations throughout the experiment. GST -3 remains constant with the shallow cosine curve of GST -6 until but increases the amplitude in the second half of the experiment. This pattern may reflect variation between parallel batches of biological replicates (see experiment design in Methods).

The cytochrome P450 superfamily of genes encodes a large and diverse group of proteins that catalyze the oxidation of organic substances including endogenous lipids and steroidal hormones and xenobiotic substances including insecticides. In *Ae. aegypti *members of the CYP9J family in particular seem to be involved in resistance to pyrethroid insecticides[27]. Figure 8 examined the abundance of transcripts from 17 members of the CYP9J family. All of these except CYP9J32 [VectorBase: AAEL008846] are located in a single cluster on chromosome 3 in the *Ae. aegypti *genome [51,27]. CYP9J19 [VectorBase:AAEL006810], CYP9J22 [VectorBase:AAEL006802] and CYP9J27b [VectorBase:AAEL014616] are represented by two oligonucleotide probes each and CYP9J27a and b are different but orthologous genes. Figure 8A demonstrates co-oscillation of CYP9J10 [VectorBase:AAEL006798], CYP9J19(478) [VectorBase:AAEL006810], CYP9J22(703) [VectorBase:AAEL006802], CYP9J24 [VectorBase:AAEL014613] and CYP9J27 VectorBase:AAEL014616] while 8B shows co-oscillations in CYP9J9 [VectorBase:AAEL006793], CYP9J22(470) [VectorBase:AAEL006802], CYP9J23 [VectorBase:AAEL014615], CYP9J27(99) [VectorBase:AAEL014616], CYP9J26 [VectorBase:AAEL014609], CYP9J27 VectorBase:AAEL014616] and CYP9J28 [VectorBase:AAEL014617] but shifted by 8 hours from the genes in 8A. 8C shows co-oscillations in CYP9J8 [VectorBase:AAEL006811], CYP9J20 [VectorBase:AAEL006814] and CYP9J29 [VectorBase:AAEL014610] but shifted by 8 hours from the seven genes in 8B and 16 hours from the genes in 8A. 8D shows that CYP9J7 [VectorBase:AAEL014606], CYP9J18 [VectorBase:AAEL006804], CYP9J19(695) [VectorBase:AAEL006810], CYP9J30 [VectorBase:AAEL014603] and CYP9J32 [VectorBase:AAEL008846] do not have an obvious oscillating pattern. However, the increase and decline pattern in one of two concatenated profiles might be indicative of a weak oscillation obscured by stochastic noise. We are uncertain as to how these oscillations affect insecticide resistance. Metabolic resistance is closely related to detoxification pathways which in turn are interlinked with oxidative phosphorylation. The oscillating pattern of oxidative phosphorylation gene expression may modulate the mosquito's ability to withstand insecticide exposure and suggests the interesting possibility that susceptibility to insecticides may show diurnal fluctuations.

The purpose of these experiments was to document that diurnal fluctuations occur in the heads of female mosquitoes. Having demonstrated these diurnal patterns; a whole series of additional questions arise. Are there diurnal fluctuations in gene expression in other adult tissues and organs? Are diurnal fluctuations seen in the developing embryo? Do they occur in larvae? How do the oscillations relate to chitin deposition and sclerotization in the cuticle? How does moulting affect oscillations? Is moulting coordinated by oscillations in different tissues? Are oscillations coordinated among different tissues? How do these oscillations respond to major perturbations in the mosquitoes environment? Specifically, how do diurnal fluctuations respond to a blood meal in the female? Are there oscillations in the production of vitellogenins (egg yolk precursor glycolipoproteins) that arise following a bloodmeal? Similarly, how are these perturbations influenced by oviposition? Are diurnal fluctuations in cytochrome P450s and Glutathione-S-Transferases perturbed by exposure to xenobiotics? If so, how soon after exposure? Are innate immunity pathways affected by the ingestion or exposure to pathogens? How does mating affect diurnal fluctuations? How are oscillations affected by lengthening or shortening the photoperiod away from the current 12:12 pattern? Due to the limitations of our incubator, a crepuscular period prior to and following the scotophase could not be included. Are the observed oscillations affected by the lack of a crepuscular period? These questions all require very specific experimental designs that are far beyond the scope or intention of the current study.

In this study we have made a first attempt to identify the baseline rhythms and co-oscillation patterns in genes in the female head beyond the basic circadian molecular clock. Due to the noisy nature of high-throughput microarray expression analysis we cannot observe a baseline rhythm in abundance of every detected transcript. However, we do observe baseline oscillation in majority of genes and thus it can be reasonably extrapolated that rhythmicity permeates every biological pathway in mosquitoes. We hypothesize that this is orchestrating changes in both mosquito physiology and behavior. Apart from the technical challenges of microarray analysis this study is limited in resolution ability by the low sampling rate of circadian time series. We could only distinguish as many as four discrete phases. The sampling rate determines the precision with which one can identify the phase of oscillation and superimpose the timing of expression of inter-dependent genes in biological pathways. We hope to intensify sampling rates in future studies.

## Conclusions

The utility of understanding the timeline of gene expression throughout the entire transcriptome is not an academic exercise limited to understanding circadian behavior. Most transcriptional experimental designs involve comparisons of strains or of individuals from a strain that exhibit different phenotypes. Implicit in these comparisons is the assumption that transcript abundance is constant within strains and individuals. If this assumption is invalid these experimental designs may have a low power to detect true differences in gene abundance. Furthermore, considered in the broader context of gene interaction networks, timeline expression can help the reverse-engineering of biological pathways, identify alternative transcripts and potential drug targets. These results are the first step towards understanding the structure and orchestration of molecular processes and gene functions in *Ae. aegypti *in relation to the dimension of time.

## Methods

### Mosquito processing

Eggs were hatched from each of 11 field collections made in 2006 from the state of Chiapas in Mexico. Cities sampled were Ciudad Hidalgo, Motozintla, Rio Florido, Puerto Chiapas, Mazatán, Huehuetán, Huixtla, Escuintla, Mapastepec, Pijijiapan, and Mixtla. The F_5 _generation of each colony was used and 600 first instar larvae from each collection were hatched in water that had been autoclaved and cooled in sealed bottles at room temperature to promote uniform hatching time. Equal numbers of larvae from the 11 collections were mixed and reared at a density of 200 mosquitoes in 2 liters of water in Pyrex^® ^4 liter autoclaved baking dishes (33 containers total). The larvae were maintained at a constant 28°C and a 12:12 photoperiod. Liver powder suspension (1 mL of a 10% (w/v) solution) was provided in the morning on each day and water was added back to maintain a constant volume.

Pupae first appeared four days after being counted into the Pyrex^® ^dishes. However, no pupae were collected for transcriptional analyses until day 8 when a large number of female pupae became available. We removed and counted all pupae into two plastic 500 mL beakers. Each beaker was placed into one of two 2' × 2' × 2' cages that were treated as biological replicates. In this context "biological replicate" means replicated extraction of biological material from separate batches of mosquitoes, but not necessarily hatched from a separate batch of eggs at a different time. Assuming an even sex ratio and allowing for daily mortality, 1,170 mosquitoes were introduced as pupae into each cage to obtain ~585 adult females.

Adult mosquitoes were maintained at a constant 28°C with 80% relative humidity, and a 12:12 photoperiod. The *light safe *incubator was locked during the 12 hour scotophase. Exactly 24 hours after placing the adults into the cage, 30 females were aspirated from replicate cage #1 into a small 500 mL cardboard carton and rapidly killed in a -80°C freezer. The heads of mosquitoes were individually removed with a scalpel and transferred with forceps into a 1.5 mL Diethylpyrocarbonate (DEPC) treated microcentrifuge tube labeled "24.1" and 100 μL of RNA *later*^® ^were added. These heads were immediately homogenized with a DEPC treated 500 μL Kontes Pellet Pestle^® ^and the volume was brought up to 500 μL with RNA *later*^®^. Contents were further homogenized until no large pieces of tissue were visible. The tube was then returned to a -80°C freezer. All steps were repeated for the second replicate cage, except that the tube was labeled "24.2" This entire process was repeated at 72, 76, 80, 84, 88, 92, 96, 100 and 104 hours after placing the adults into the cage. At the end of the experiment two replicate pools of female heads had been collected at each of 10 time points.

### RNA isolation, cRNA synthesis, amplification and labeling

The RNeasy Midi-kit (QIAGEN Inc. Valencia, CA) was used to isolate total RNA according to manufacturer's instructions. RNA was eluted in 150 μL DEPC--ddH2O into a 1.5 mL DEPC-treated tube. RNA concentration was read on a Nanodrop^® ^spectrophotometer and the tube was maintained at -80°C. The Agilent RNA Spike-In^® ^kit provided two-colored standards in all experiments. RNA (500 ng) was placed in a 200 uL tube along with two uL of either Diluted Spike A or B and 1.2 uL of T7 Promoter Primer Mix. RNA from 24 hour old heads was labeled with Cyanine 5-CTP while RNA from all heads collected at later time points were labeled with Cyanine 3-CTP using the Agilent Technologies^® ^low input linear amplification RNA labeling kit according to the manufacturer's instructions. The RNeasy Mini-kit (QIAGEN Inc.) was used to purify the labeled/amplified cRNA. The purified cRNA was eluted in 30 μL RNase-free water and quantified in pmol/μL using a Nanodrop^® ^spectrophotometer. We did not proceed to the hybridization steps if the total yield was < 825 ng or if the specific activity was < 8.0 pmol Cy3 or Cy5 per μg cRNA.

### Hybridization

The Agilent^® ^*Aedes aegypti *microarray described in Xi et al [33] and Nene et al [16] was used. Each array contained 45,220 features, 43,803 of which correspond to 21,494 unique *Ae. aegypti *oligonucleotides replicated twice (20,692), thrice (793) or four times (10). The remaining features hybridized to oligonucleotides contained in the Agilent RNA Spike-In^® ^kit for quality control. Hybridizations were conducted with the Agilent Technologies^® ^In Situ Hybridization kit at 60°C according to the manufacturer's instructions. Hybridization intensities were determined with an Axon GenePix 4100AL scanner at 635 nm for Cy-5 and at 532 nm for Cy3, and images were analyzed with Gene Pix Pro 6.0 software. The quality of the hybridization was assessed with Gene Pix software for control, feature and replicate quality control software packages. Two of the 26 arrays that were processed failed to pass the quality control screens. Both were repeated from the original RNA and passed on the second attempt.

### Data pre-processing

Values from all 1,417 spots that hybridize to the probes in the Agilent^® ^RNA Spike-In kit were removed. Median background values at 635 nm were subtracted from median spot values and the same was repeated for background and spot values at 532 nm. Background corrected values were then transformed to relative intensities using the formula:

so that all Cy3 values from 72 - 104 hours were standardized against the same pool of Cy5 labelled RNA from the 24 hr timepoint. This experiment layout has been chosen to standardise the impact of technical variation on different time points. The chosen reference point is the same for all microarrays and placed far ahead of the starting point for the time series to allow sufficient numbers of genes differentially expressed in comparison to the Cy5 control at any point of the time series. Thus estimates of RNA quantities for each of the 21,494 *Ae. aegypti *gene features was estimated from 2-4 technical replicates and two biological replicates.

### Analysis of periodicity

We composed 48 h gene expression profiles out of a series of observation covering continuous 32 h period with 2 independent replicates for each time point sampled every 4 h. The technical replicates were processed as separate entities, rather than averaged. The two biological replicates we considered as two independent timelines. The first six time points of each were concatenated to construct a continuous 48 hour time line used in analysis of periodicity. This experimental design reconstructing two consecutive periods from two simultaneously processed independent timelines has been approbated in previous studies [36]. The reconstructed time series starts with the first time point at 9 am. The experiment design with numbers of replicates and time of sample collection is given in Supplemental Table 1 (see Additional File 1).

Expression profiles were smoothed using a 3^rd ^degree polynomial procedure and median-subtracted using the seven-point Savitzky-Golay algorithm [52]. To take advantage of all points in the time series a single-pass smoothing was applied in a circular manner, with the last points contributing to smoothing of the starting points. The same smoothing and median subtraction procedure was applied to all data sets. The results of application of periodicity tests to individual gene expression profiles are given in Supplemental Table 2 (see inside Additional File 1).

### Spectral Analysis

For purposes of spectral analysis, consider a series of microarray expression values for gene *x *with *N *samples of the form:

This series can be converted from time-domain, where each variable represents a measurement in time to a frequency domain using a Discrete Fourier Transform (DFT) algorithm. Frequency domain representation of the series of experiments is also known as a periodogram, which can be denoted by *I*(ω):

If a time series has a significant sinusoidal component with frequency ω ∈ [0, *π*], then the periodogram exhibits a peak at that frequency with a high probability. Conversely, if the time series is a purely random process (a.k.a "white noise"), then the plot of the periodogram against the Fourier frequencies approaches a straight line [53].

### Fisher's g-test

The significance of the observed periodicity can be estimated by Fisher's *g*-statistic, as recently recommended [54]. Fisher derived an exact test of the maximum periodogram coordinate by introducing the *g*-statistic:

where *I*(*ω*_*k*_) is a *k-*th peak of the periodogram. Large values of g indicate a non-random periodicity. We calculate the *p*-value of the test under the null hypothesis with the exact distribution of *g *using the following formula:

where *n *= [*N/*2] and *p *is the largest integer less than 1*/x*.

This algorithm closely follows the guidelines recommended for analysis of periodicities in time-series microarray data [54] except that we applied C++ code (written by and available from AP) instead of R scripts. Fisher's g-test has low power on short time series under 50 samples [55]. Attaining such series using contemporary technology would be prohibitively expensive. However, the problem can be mitigated by application of the g-test in a phase continuum setting (see below).

### Autocorrelation

For a given a discrete time series *Y *= *x*_0_, *x*_1_, *x*_2_, ...*x*_*N*-1 _the autocorrelation is simply the correlation of the expression profile against itself with a frame shift of *k *data points (where 0 ≤ *k *≤ *N*-1, often referred as the lag). For the time shift *f*, defined as *f *= *i *+ *k *if *i *+ *k *<*N *and *f *= *i *+ *k *- *N *otherwise:

For each time series we calculated the maximum positive *R(f) *among all possible phase shifts *f *and use tabulated 0.05 significance cut off values for correlation coefficient. Time series that shows significant autocorrelation *R(f) *with the lag *f *corresponding to one day (6 time points × 4 hours) are considered circadially expressed.

### Pt-test

Consider a time series *Y *= *x*_0_, *x*_1_, *x*_2_, ...*x*_*N*-1 _in which technical variation approaches or even exceeds the amplitude of periodic expression. In a very short time series stochastic noise often obscures periodicity. However, the periodic change of the base expression level can still be identified in spite of the high noise level. If the periodogram of the original time series *IY(ω) *contains a significant peak corresponding to a particular frequency (e.g. circadian) this peak results from observation in the *Y*. A random permutation would preserve the same noise level, but not the periodicity. Let *YR *be a random permutation of *Y *with a corresponding periodogram is *IR(ω)*. After applying the DFT, a periodogram *IR(ω) *would represent only the peaks occurring by chance. However it would miss the true periodic frequencies unless permutations happen to preserve the period. This could occur if, for example the rank of each point *x *in a permutated series *YR *is X_Y _± *np *where *n *is a natural number and *p *is a period corresponding to a significant peak in *IY(ω)*. To avoid random re-institution of periodicity a C++ program was written to generate *YR *by multiple shuffling of randomly selected time points *x*_*n *_⇔ *x*_*m*_, where |*n *- *m*| ≠ *p*. For each shuffle the program swaps time points from a different phase. Comparing permutations with deliberately wiped out periodicity to the original time series, we estimated whether the original order of observations minimized the overall noise. For each gene expression profile we generated two series of *min (n!,100) *random permutations. Each permutated series *YR *was transformed to the frequency domain and a single peak of the periodogram *IR(ω) *was stored. The p-value for the null-hypothesis of random nature of a particular peak of periodogram can be estimated by comparing the stored *IR(ω) *values to the observed *I(ω)*:

High *p*-values that exceeded the threshold, for example 0.05, indicate that at least 5 out of 100 random permutations of the time series produced a periodogram with the same or a higher peak, corresponding to a given periodicity. Low *p*-values indicate a significant difference between periodogram *IR(ω) *preserving circadian periodicity and randomly permutated periodogram *IY(ω) *with the same level of technical variation. This difference leads to rejection of the null-hypothesis of purely random nature of variation in the original time series *Y*.

### Phase continuum

We start with phase classification, assigning each gene a phase based on maximal correlation to an ideal cosine curve. This method is superior to assigning a phase by position of peaks only because it takes into account more data. Each profile is subjected to z-score transformation equalizing the variation between time points. Autocorrelation with circadian lag (*R_c_*) was calculated for each profile and all profiles were sorted first by phase then by descending order of *R_c_*. Concatenating all profiles of the same phase with an equalized range of variation (amplitude) we generate a continuous stream *C_ph _*of measurements containing a clear signal on one end and stochastic noise on the other. This continuum was treated with a low-pass frequency filter and polynomial smoothing [52]. As discussed in the original publication of the method, some digital filters can artificially modulate the expression profile [40]. For this reason we have limited the choice of filters to a few least likely to propagate the oscillation along the phase continuum. We analyzed each phase fraction separately to detect the point at which circadian signal deteriorates beyond a p = 0.05 cutoff. A window W moving along the stream is tested for periodicity using one of the previously described tests. Once the point at which *I_w _*does not differ significantly from a random periodogram *I_wr_*, we counted all original gene expression profiles that had circadian signal above the established cutoff [40]. Here we applied the frame length 5 (testing 5 genes or 60 timepoints at a time, the recommended minimal length for g-test power) for Fisher's g-test and frame length 3 for Permutation and Autocorrelation tests.

### False Discovery Rate analysis

This methodology is often applied to reduce the number of false-positive results. It is based in the assumption of independent or mildly dependent [56] hypothesis testing. However, in the case of testing timeline expression profiles for periodicity, independence cannot be assumed. First, the pattern of circadian oscillation is obvious in the great majority of expression profiles (see Figure 1, for example). Second, an analysis of correlations with phase shift (also used to identify phase groups) confirms high correlation of nearly all profiles to common cosine curves. Third, living cells are known to have more than one oscillator, but these oscillators are normally synchronized to the rhythm of the circadian molecular clock, active in peripheral tissues. When testing individual expression profiles for periodicity we are looking for manifestation of the same factor, hence not an independent hypothesis. For these reasons FDR correction was not applied to reduce the number of detected oscillating genes. In earlier publications exploring this methodology independent validation of expression profiles confirmed oscillation pattern for multiple genes that did not pass the periodicity test, with or without FDR adjustment [36].

### Biological Pathway analysis

Functional annotation of *A. aegypti *transcripts targeted by microarray followed the annotation of nearest orthology in the *D. melanogaster *genome. Information on gene interaction and charts for biological pathways specific to *A. aegypti *has been extracted from the Kyoto Encyclopedia of Genes and Genomes (KEGG). Correspondence between KEGG genes and *A. aegypti *probe sequences has been verified by the rapid BLAT [57] search.

### Real Time PCR analysis

Microarray results were tested for selected genes using real time PCR amplification of 2 ul of cDNA, and 10 μm of each primer with SYBR green detection in a Bio-Rad iCYCLER using the IQ SYBR Green Supermix (Bio-Rad, Hercules, CA 94547). The cycling conditions were Step 1: 50.0°C for 2 min Step 2: 95.0°C for 10 min; Step 3: 95.0°C for 10 seconds Step 4: 60.0°C for 20 seconds, Step 5: 72.0°C for 10 seconds. Repeat steps 3-5, 40 times. This was followed by a melting curve analysis with 55 points collected between 68.0°C and 95.0°C.

### Heatmap visualization

A heatmap was constructed to represent gene expression profiles in 72 hour-104 old female mosquito heads wherein all twelve timepoints are represented as columns along the abscissa. Each line on the ordinate corresponds to the M-values for a particular gene feature and appear as shades of red to black to green. Bright red indicates large positive M-values (M_24 _> M_t_, where t = 1,2,....,12), while black values indicate M_24 _≃ M_t. _and bright green indicates large negative M-values (M_24 _< M_t_).

Each column is further vertically subdivided into four "same-phase" groups. Each gene feature was assigned to a group that contained gene features with the same-phase as explained above. Within each group, gene expression profiles were sorted and stacked on top of each other so that most clearly oscillating (i.e. highest signal to noise ratio) profiles are on top and the least periodic profiles are at the bottom of each group. All expression profiles were tested for periodicity by autocorrelation test and sorted in order of decreasing correlation between an early time and a second time 24 hours later. Additional explanation of the algorithm for generating gene expression heatmap is given in Supplemental Figure 1 (see inside Additional File 1).

## Authors' contributions

AP analyzed the data, wrote the paper; GR performed experiments; KS performed the experiments; JB performed the experiments; ES designed the study, wrote the paper; JC designed the study, wrote the paper; WB designed the study, wrote the paper. All authors read and approved the final manuscript.

## Supplementary Material

Additional file 1 **Supplementary Information**. This is a zip archive file that contains Supplemental Table 1 (MS Excel file), Supplemental Table 2 (MS Excel file) and Supplemental Figure 1 (Adobe PDF file). Supplemental Table 1 reports the experiment design for sample collection with date, time, pooling and replication information. Supplemental Table 2 reports the results of straight application of periodicity tests to reconstructed 48 h expression profiles (see Methods). Supplemental Figure 1 illustrates the process of generation of circadian expression heat map (presented in Figure 1). Click here for file
