# Supplementary material for: Rhythms and synchronization patterns in gene expression in the Aedes aegypti mosquito
Source: BMC Genomics. 2011 Mar 17;12:153. doi: 10.1186/1471-2164-12-153 (PMC3072344; doi:10.1186/1471-2164-12-153)
Supplement: Additional file 1 — Supplementary Information. This is a zip archive file that contains Supplemental Table 1 (MS Excel file), Supplemental Table 2 (MS Excel file) and Supplemental Figure 1 (Adobe PDF file). Supplemental Table 1 reports the experiment design for sample collection with date, time, pooling and replication information. Supplemental Table 2 reports the results of straight application of periodicity tests to reconstructed 48 h expression profiles (see Methods). Supplemental Figure 1 illustrates the process of generation of circadian expression heat map (presented in Figure 1). [file 1471-2164-12-153-S1.ZIP › SupplementalFigure1.pdf]

Explanation of Heatmap Generation (Figure 1)

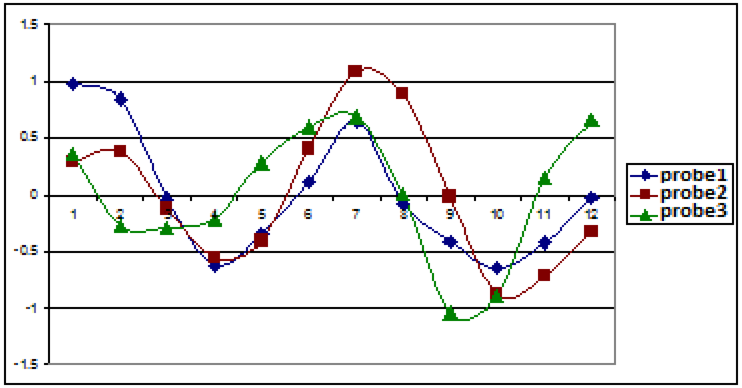

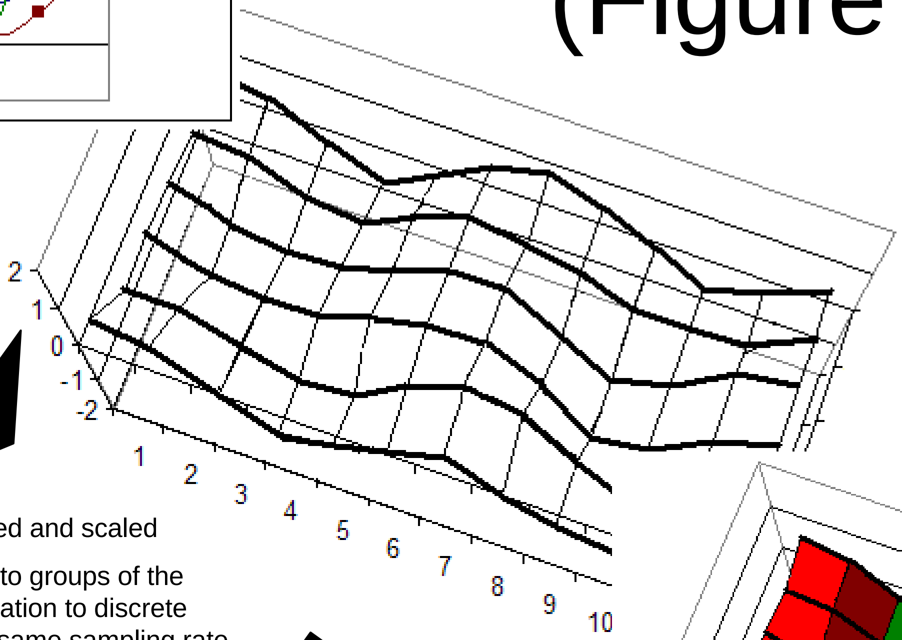

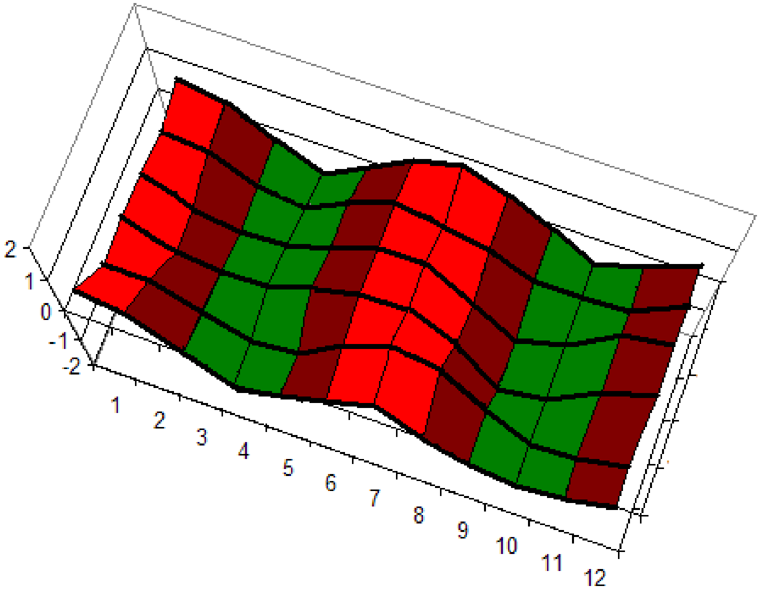

1. Expression profiles are normalized and scaled
2. Expression profiles are binned into groups of the same phase (estimated by correlation to discrete cosine curve generated with the same sampling rate and different phase shifts). Time series of 12 points can be separated into four distinct phases
3. In each phase group genes are arranged in the order of decreasing signal to noise ratio
4. Profiles are stacked in that order with least noisy on top and most noisy at the bottom
5. Peak expression is colored red, low expression green with color range in between
6. Each discrete phase group is charted separately and phase groups are placed on top of each other
